# Fotagliptin monotherapy with alogliptin as an active comparator in patients with uncontrolled type 2 diabetes mellitus: a randomized, multicenter, double-blind, placebo-controlled, phase 3 trial

**DOI:** 10.1186/s12916-023-03089-x

**Published:** 2023-10-09

**Authors:** Mingtong Xu, Kan Sun, Wenjie Xu, Chuan Wang, Dewen Yan, Shu Li, Li Cong, Yinzhen Pi, Weihong Song, Qingyuan Sun, Rijun Xiao, Weixia Peng, Jianping Wang, Hui Peng, Yawei Zhang, Peng Duan, Meiying Zhang, Jianying Liu, Qingmei Huang, Xuefeng Li, Yan Bao, Tianshu Zeng, Kun Wang, Li Qin, Chaoming Wu, Chunying Deng, Chenghu Huang, Shuang Yan, Wei Zhang, Meizi Li, Li Sun, Yanjun Wang, HongMei Li, Guang Wang, Shuguang Pang, Xianling Zheng, Haifang Wang, Fujun Wang, Xiuhai Su, Yujin Ma, Wei Zhang, Ziling Li, Zuoling Xie, Ning Xu, Lin Ni, Li Zhang, Xiangqun Deng, Tianrong Pan, Qijuan Dong, Xiaohong Wu, Xingping Shen, Xin Zhang, Qijing Zou, Chengxia Jiang, Jue Xi, Jianhua Ma, Jingchao Sun, Li Yan

**Affiliations:** 1 Sun Yat-Sen Memorial Hospital, Sun Yat-Sen University, Guangzhou, China; 2 Shenzhen Salubris Pharmaceuticals Co., Ltd, Shenzhen, China; 3 Shenzhen Second People’s Hospital, Shenzhen, China; 4 Huizhou Municipal Central Hospital, Huizhou, China; 5 The Fifth Affiliated Hospital of Sun Yat-Sen University, Zhuhai, China; 6 The First Hospital of Changsha, Changsha, China; 7 Chenzhou First People’s Hospital, Chenzhou, China; 8 Yueyang Central Hospital, Yueyang, China; 9 The First Peole’s Hospital of Xiangtan City, Xiangtan, China; 10 Yiyang Central Hospital, Yiyang, China; 11 The Second Affiliated Hospital of the University of South China, Hengyang, China; 12 Yichun People’s Hospital, The Affiliated Hospital of Yichun University, Yichun, China; 13 Pingxiang People’s Hospital, Pingxiang, China; 14 The People’s Hospital of Nanchang, The Third Hospital of Nanchang, Nanchang, China; 15 The Second Affiliated Hospital of Nanchang University, Nanchang, China; 16 The First Affiliated Hospital of Nanchang University, Nanchang, China; 17 Xinyu People’s Hospital, Xinyu, China; 18 Taihe Hospital, Affilited Hospital of Hubei University of Medicine, Shiyan, China; 19 Renmin Hospital of Wuhan University, Wuhan, China; 20 Union Hospital, Tongji Medical College, Huazhong University of Science and Technology, Wuhan, China; 21 Nanjing Jiangning Hospital, The Affiliated Jiangning Hospital of Nanjing Medical University, Nanjing, China; 22 Chongming Branch, Xinhua Hospital Affiliated to Shanghai Jiaotong University School of Medicine, Shanghai, China; 23 The 2nd School of Medicine, WMU, The 2nd Affiliated Hospital and Yuying Children’s Hospital of WMU, Wenzhou, China; 24 Zigong Fourth People’s Hospital, Zigong, China; 25 Bishan Hospital of Chongqing, Bishan Hospital of Chongqing Medical University, Chongqing, China; 26 Fourth affiliated hospital of Harbin Medical University, Harbin, China; 27 The First Hospital of Qiqihar, Qiqihar, China; 28 The Affiliated Hospital of Yanbian University, Yanbian, China; 29 Siping Central People’s Hospital, Siping, China; 30 The Second Norman Bethune Hospital of Jilin University, Jilin, China; 31 Emergency General Hospital, Beijing, China; 32 Beijing Chao-Yang Hospital, Capital Medicine University, Beijing, China; 33 Jinan Central Hospital, Central Hospital Affiliated to Shandong First Medical University, Jinan, China; 34 Handan Central Hospital, Handan, China; 35 Handan First Hospital, Handan, China; 36 The Fourth Hospital of He Bei Medical University, Shijiazhuang, China; 37 Cangzhou Hospital of Integrated TCM-WM Heibei, Cangzhou, China; 38 The First Affiliated Hospital and College of Clinical Medicine, Henan University of Science and Technology, Luoyang, China; 39 Puyang Oilfield General Hospital, Puyang, China; 40 Inner Mongolia Baogang Hospita, Baotou, China; 41 Zhongda Hospital Southeast University, Nanjing, China; 42 The First People’s Hospital of Lianyungang, Lianyungang, China; 43 The First People’s Hospital of Huzhou, The First Affiliated Hospital of Huzhou Teacher College, Huzhou, China; 44 Hainan Third People’s Hospital, Sanya, China; 45 Wuhan Third Hospital, Wuhan, China; 46 The Second Hospital of Anhui Medical University, Hefei, China; 47 People’s Hospital of Zhengzhou, People’s Hospital of Henan University of Chinese Medicine, Zhengzhou, China; 48 Zhejiang Provincial People’s Hospital, People’s Hospital of Hangzhou Medical College, Hangzhou, China; 49 Zhongshan Hospital Xiamen University, Xiamen, China; 50 Genertec Liaoyou Gem Flowe Hospital, Panjin, China; 51 The Central Hospital of Yougzhou, Yongzhou, China; 52 The First People’s Hospital of Zunyi, Zunyi, China; 53 The Affiliated Hospital of Xuzhou Medical University, Xuzhou, China; 54 Nanjing First Hospital, Nanjing, China

**Keywords:** Dipeptidyl peptidase-4 inhibitors, Fotagliptin, Type 2 diabetes mellitus, HbA1c

## Abstract

**Background:**

Dipeptidyl peptidase-4 inhibitors (DPP-4i) have become firmly established in treatment algorithms and national guidelines for improving glycemic control in type 2 diabetes mellitus (T2DM).To report the findings from a multicenter, randomized, double-blind, placebo-controlled phase 3 clinical trial, which was designed to assess the efficacy and safety of a novel DPP-4 inhibitor fotagliptin in treatment-naive patients with T2DM.

**Methods:**

Patients with T2DM were randomized to receive fotagliptin (*n* = 230), alogliptin (*n* = 113) or placebo (*n* = 115) at a 2:1:1 ratio for 24 weeks of double-blind treatment period, followed by an open-label treatment period, making up a total of 52 weeks. The primary efficacy endpoint was to determine the superiority of fotagliptin over placebo in the change of HbA1c from baseline to Week 24. All serious or significant adverse events were recorded.

**Results:**

After 24 weeks, mean decreases in HbA1c from baseline were -0.70% for fotagliptin, -0.72% for alogliptin and -0.26% for placebo. Estimated mean treatment differences in HbA1c were -0.44% (95% confidence interval [CI]: -0.62% to -0.27%) for fotagliptin versus placebo, and -0.46% (95% CI: -0.67% to -0.26%) for alogliptin versus placebo, and 0.02% (95%CI: -0.16% to 0.19%; upper limit of 95%CI < margin of 0.4%) for fotagliptin versus alogliptin. So fotagliptin was non-inferior to alogliptin. Compared with subjects with placebo (15.5%), significantly more patients with fotagliptin (37.0%) and alogliptin (35.5%) achieved HbA1c < 7.0% after 24 weeks of treatment. During the whole 52 weeks of treatment, the overall incidence of hypoglycemia was low for both of the fotagliptin and alogliptin groups (1.0% each). No drug-related serious adverse events were observed in any treatment group.

**Conclusions:**

In summary, the study demonstrated improvement in glycemic control and a favorable safety profile for fotagliptin in treatment-naive patients with T2DM.

**Trial registration:**

ClinicalTrail.gov NCT05782192.

**Supplementary Information:**

The online version contains supplementary material available at 10.1186/s12916-023-03089-x.

## Background

The prevalence of type 2 diabetes mellitus (T2DM) has increased markedly with an estimated number of 347 million individuals worldwide in 2008, and forecasted to increase to 7079 individuals per 100, 000 by 2030 [1]. For T2DM management, a lot of guidelines have recommended metformin as a first-line therapy in combination with healthy diet and exercise, and provided recommendations on second-line therapies when metformin is unable to achieve or maintain long-term glycemic control [2–4]. However, the selection of second-line therapies for T2DM is challenging. Although algorithms provide evidence-based principles and guidelines, several factors should be taken into consideration to determine the optimum approach, such as patient age, individual compliance, financial conditions and diabetes complications [5, 6].

Dipeptidyl peptidase-4 inhibitors (DPP-4i) could improve glycemic control by preventing the rapid degradation of incretin hormones and inhibiting glucagon secretion [7]. With a low risk of hypoglycemia and no weight gain, nearly 0.7% reduction in HbA1c was reported when other DPP-4 inhibitors were given either alone or in combination with metformin for the treatment of T2DM patients [8–10]. Fotagliptin (Salubris Pharmaceuticals, Shenzhen, China) is a selective, once-daily, novel DPP-4 inhibitor approved for glycemic management of T2DM. Preclinical pharmacological studies showed that fotagliptin could inhibit DPP-4 with IC_50_of 2.27 nM [11]. Fotagliptin was not primary metabolized by cytochrome P450 enzymes. There were 2 major metabolites, M1 had no inhibitory effect on DPP-4, M2-1 had slight inhibition [12]. The efficacy and safety profiles of fotagliptin have been characterized in previous studies. Fotagliptin exhibited favorable pharmacokinetic results as it can achieve high and steady DPP-4 inhibition. Fotagliptin was rapidly absorbed, which t_max_ was obtained at 1–2 h in T2DM patients, also enabling a maximum inhibition of DPP-4 within 1–2 h post administration [12, 13]. Recently, a phase 1 clinical trial has been conducted in fourteen eligible Chinese patients with T2DM, which showed that fotagliptin was safe and well tolerated [12]. In a phase 2 clinical study, patients who were treated with fotagliptin monotherapy of 6 mg, 12 mg and 24 mg once a day for 12 weeks showed significant improvement in the HbA1c control as compared with placebo. The hypoglycemic effect increased with the treatment duration, and the biggest decrease in HbA1c was observed in the 12 mg fotagliptin group.

This is the first phase 3 randomized, double-blind, placebo-controlled clinical study to evaluate the efficacy and long-term safety of fotagliptin in treatment-naive T2DM patients uncontrolled with diet and exercise intervention, comprising 24 weeks of double-blind treatment period followed by an open-label treatment period, making up a total of 52 weeks.

## Methods

### Study design and randomization

Patients with type 2 diabetes mellitus (T2DM) were randomized to receive fotagliptin, alogliptin or placebo. Subjects who met the inclusion criteria would enter the 4 weeks of placebo run-in period. At the end of the run-in period, a baseline enrollment evaluation was performed. Upon evaluation, all eligible subjects would enter the 24 weeks of double-blind treatment period and were randomized into the fotagliptin group (12 mg once daily) or alogliptin group (25 mg once daily) or placebo group at a 2:1:1 ratio. Randomization and drug dispensation were performed with an interactive web response system (IWRS;eBalance version 5.3.7). A stratified randomization method with the permuted block randomization algorithm was used. The blocks were dynamically allocated to each site and stratum from the randomization list. A unique ID number was provided by the vendor and marked on the medication box. Using central randomization, randomization codes were assigned to eligible participants by the IWRS system based on stratification factors (baseline HbA1c level < 8.5% or ≥ 8.5%) and the block size. After 24 weeks of double-blind treatment, subjects would enter the extended open-label treatment period. Subjects in the placebo group were to be switched to fotagliptin (12 mg once daily) treatment, while patients in the fotagliptin and alogliptin groups continued the same treatment until the end of the whole 52 weeks.

The trial was conducted in accordance with the *Declaration of Helsinki* and *Good Clinical Practice* principles. The protocol was approved by the independent institutional review boards or ethics committees of each site that participated in the study. All investigational medicinal products (IMP) and matching placebo were provided by Shenzhen Salubris Pharmaceuticals Co., Ltd. The written informed consent was obtained from all the patients before the implementation of study procedures. The study was registered on ClinicalTrails. gov, number NCT05782192.

### Population

Subjects who were 18–75 years old and with previously untreated T2DM (no oral or injected anti-diabetes treatment before 8 weeks of randomisation) were eligible for screening. After a 4-week diet and exercise run-in period, eligible study participants with poor glycemic control (hemoglobin A1c [HbA1c] values of 7.5% to 10.5% inclusive and fasting blood glucose [FBG] ≤ 13.9 mmol/L) were included in the trail. Main exclusion criteria were: treatment with any antihyperglycaemic medication within the run-in period of the trial; hypoglycaemic unawareness or recurrent severe hypoglycaemia; anaphylactic reaction or contraindication to any IMP or placebo; impaired renal or hepatic function; acute or severe chronic complications of diabetes. The full inclusion and exclusion criteria are avaliable in Supplementary table 1.

### Outcomes and assessments

The primary efficacy endpoint was to evaluate the HbA1c from baseline to Week 24 in T2DM patients treated with fotagliptin 12 mg/day, in parallel control with alogliptin and placebo. The secondary endpoints were change in HbA1c from baseline to Week 52; change in FBG from baseline to Week 52; occurrence of hypoglycemia from baseline to Week 52; change in bodyweight from baseline to Week 52. All adverse events were followed up by investigators and have been sufficiently characterised. The following adverse events were to be reported: serious adverse events occured in more than 2% of the patients in either treatment group; any adverse events occurred in more than 5% patients in either treatment group; any other safety events of special interest.

### Statistical analysis

The primary objective was to determine the superiority of fotagliptin over placebo in the change of HbA1c from baseline to Week 24, and the two-sided test of superiority/non-inferiority design was adopted with α = 0.05 and β = 0.20. According to the phase 3 clinical trial results of other drugs in the same class, it is estimated that the primary efficacy endpoint of HbA1c difference between fotagliptin and placebo is 0.45% [14], assuming a standard deviation of 1.0%. The non-inferiority limit between fotagliptin and alogliptin is 0.4. With the 2:1:1 ratio of fotagliptin group, alogliptin group and placebo group, the sample size of the three groups was estimated to be 160, 80 and 80 patients, respectively. Considering the rate of loss to follow-up and to obtain safety data through Week 52, the sample size was estimated to be 224, 112 and 112 patients in the fotagliptin group, alogliptin group and placebo group, respectively.

The primary analysis was performed in the full analysis set (FAS), which received at least one dose of the study drug and had at least one posttreatment measurement of the endpoint during the double-blind treatment period. Missing data were imputed using the last observation carried forward (LOCF). HbA1c values at follow-up visits of subjects received rescue were treated as missing values. As a dependent variable, the change of HbA1c from baseline to Week 24 was analyzed using the analysis of covariance (ANCOVA) model. With the treatment group as the fixed effect and the baseline HbA1c as a linear covariate, we assessed the least squares mean and standard error of the primary efficacy endpoint of each treatment group, as well as the differences of the primary efficacy endpoint and its standard error or 95% confidence interval between groups.

Descriptive statistics (frequency and percentage) were used to summarize the patient demographics, incidence of adverse events and hypoglycemic episodes by treatment group. Continuous variables, such as physical examination and clinical laboratory evaluations, were summarized by means ± standard deviation (SD) for normally distributed data or medians (interquartile ranges) for non-normally distributed data. All statistical tests were two-sided, with *P* value < 0.05 considered as statistically significant. Statistical analysis was performed using SAS version 9.4 (SAS Institute Inc, Cary, NC, USA).

## Results

**Fig. 1:**
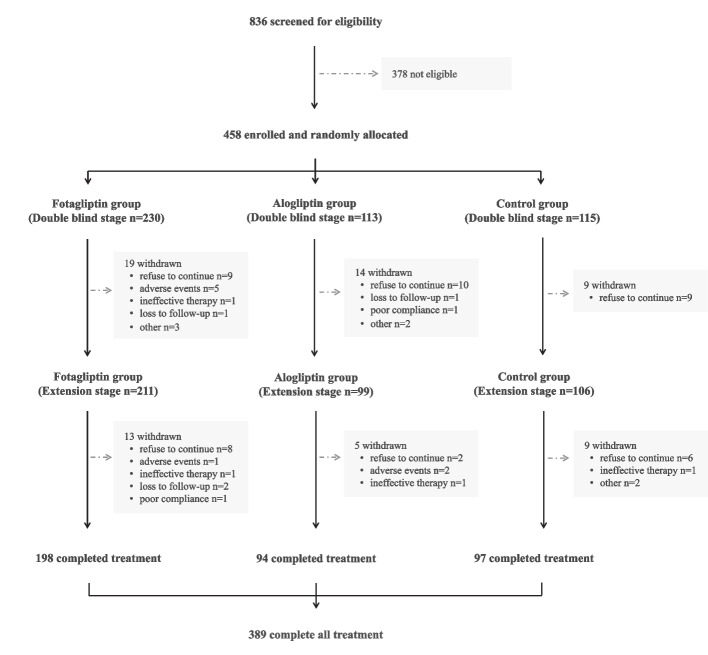
Study patient disposition

In all, a total of 836 patients with T2DM from 56 sites were screened for eligibility. After the run-in period, 458 patients were randomized in the double-blind period as follows: 230 patients in the fotagliptin group, 113 patients in the alogliptin group, and 115 patients in the placebo group. In the fotagliptin group, alogliptin group and placebo group, 19 (8.2%), 14 (12.3%), and 9 (7.8%) patients prematurely discontinued the study and the most common reason for study discontinuation was voluntary withdrawal (9, 10, and 9 patients, respectively). There were 91% (416/458) patients compliant with the study drug dosing throughout the double-blind period while 94% (389/416) of these patients completed the whole 52-week treatment period. Details of the patients’ disposition are showed in Fig. 1. The FAS included 450 patients. At baseline, demographic and clinical characteristics were well balanced across treatment groups. There were no differences across the fotagliptin, alogliptin and placebo groups in mean HbA1c and other glycemic indicators (Table 1).

**Table 1:** Demographic and clinical characteristics of the patients at baseline

	Fotagliptin group (*n* = 227)	Alogliptin group (*n* = 110)	Placebo group (*n* = 113)	P
Age (years)	53.0 (9.03)	53.7 (10.37)	54.2 (9.75)	0.4508
Male [n (%)]	140 (61.7)	70 (63.6)	72 (63.7)	0.9080
BMI (kg/m2)	25.67 (3.12)	25.69 (3.07)	25.65 (2.91)	0.9911
Weight (kg)	70.77 (11.18)	69.76 (11.69)	69.20 (11.45)	0.2494
SBP (mmHg)	124.9 (11.6)	126.4 (12.5)	125.3 (12.0)	0.5502
DBP (mmHg)	81.1 (7.9)	81.9 (8.0)	81.0 (7.4)	0.7377
eGFR (ml/min per 1.73 m2)	104.92 (11.45)	101.84 (14.19)	102.18 (13.98)	0.0276
ALT (U/L)	23.0 (17.0, 33.0)	21.9 (15.0, 33.0)	24.0 (16.0, 35.3)	0.3979
AST (U/L)	20.0 (16.3, 25.7)	21.0 (16.0, 27.0)	21.0 (17.0, 27.0)	0.5847
Duration of diabetes (months)	11.00 (1.80,36.50)	13.20 (2.70,38.60)	19.25 (2.35,34.85)	0.6666
FPG (mmol/L)	9.34 (1.79)	9.40 (2.06)	9.47 (1.79)	0.8566
HbA1c (%)	8.08 (0.73)	8.10 (0.74)	8.12 (0.70)	0.8495
Fasting insulin (μIU/ml)	10.67 (7.55,15.80)	10.36 (6.61,16.32)	10.58 (7.19,15.04)	0.7627
Fasting C-peptide (ng/ml)	2.49 (2.01,3.14)	2.37 (1.96,3.07)	2.51 (1.93,3.27)	0.5884
HOMA-β	35.62 (23.40,53.77)	33.14 (20.46,57.54)	32.91 (22.89,48.21)	0.4523
HOMA-IR	4.45 (3.10,6.93)	4.61 (2.66,6.41)	4.47 (2.94,7.03)	0.8199

1 Data were means ± SD or medians (interquartile ranges) for skewed variables or numbers (proportions) for categorical variables

2 P for trend was calculated for the linear regression analysis tests across the groups. *P* values were for the ANOVA or χ2 analyses across the groups

3 *BMI* body mass index, *SBP* systolic blood pressure, *DBP* diastolic blood pressure, *eGFR* estimated glomerular filtration rate, *FPG* fasting plasma glucose, *γ-GGT* γ-glutamyltransferase

**Fig. 2:**
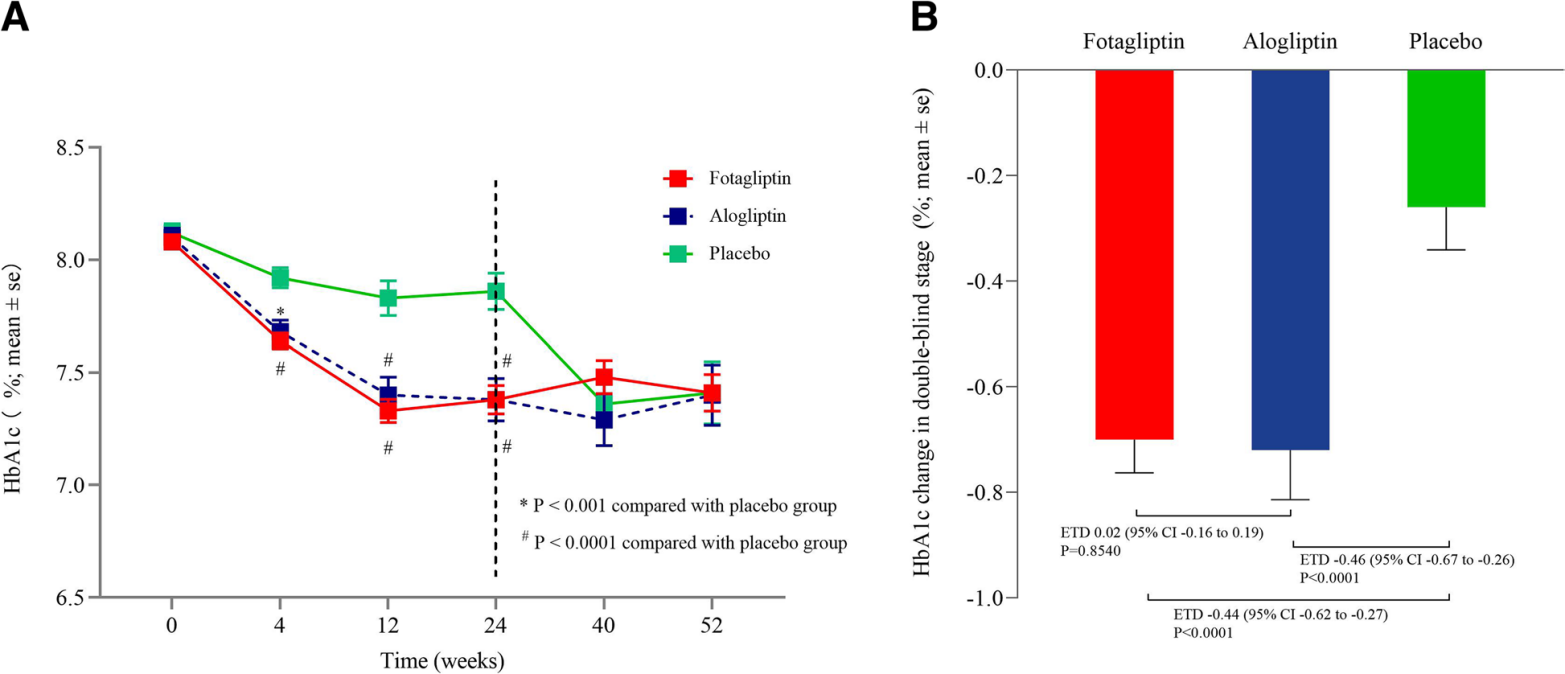
Differences in primary clinical end points (Full-analysis-set). **A** HbA1c values. **B** HbA1c values change in double-blind stage. Data are mean and error bars are SEs. The ETD and corresponding 95% CI were estimated using an ANCOVA in the FAS. Last observation carried forward imputation was used for missing values

The primary efficacy endpoint was change from baseline in HbA1c at Week 24. As shown in Fig. 2A, HbA1c reductions were superior with fotagliptin and alogliptin versus placebo at Week 24 (All *P* values < 0.0001). Mean decreases in HbA1c from baseline to Week 24 were -0.70% for fotagliptin, -0.72% for alogliptin and -0.26% for placebo (Fig. 2B). Estimated mean treatment differences were -0.44% (95% CI: -0.62% to -0.27%) for fotagliptin versus placebo, and -0.46% (95% CI: -0.67% to -0.26%) for alogliptin versus placebo at Week 24. Fotagliptin was also non-inferior to alogliptin, as estimated mean treatment difference of fotagliptin vs alogliptin was 0.02% (95%CI: -0.16% to 0.19%; upper limit of 95%CI < margin of 0.4%). Results from sensitivity analysis supported the results of confirmatory analysis (Supplementary Figs. 2, 3, 4). A total of 20 (4.4%) subjects used rescue therapy with biguanides in the double-blind period, including 5 patients (2.2%) in the fotagliptin group, 6 patients (5.5%) in the alogliptin group, and 9 patients (8.0%) in the placebo group. In the extended treatment period, 92% (97/106) subjects in the placebo group used fotagliptin at a dose of 12 mg once daily and there were no differences in HbA1c from baseline to Week 52 across treatment groups (Fig. 2A).

**Fig. 3:**
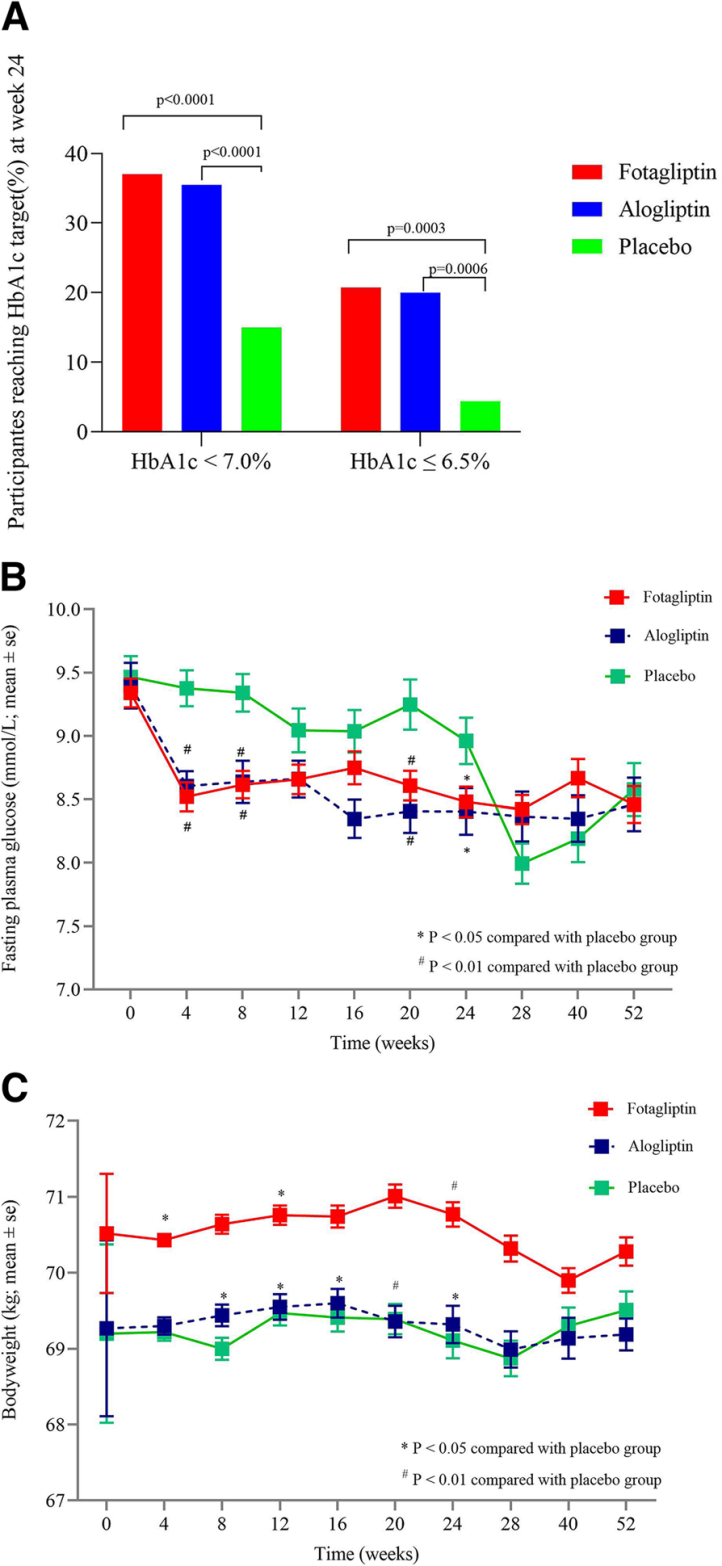
Differences in secondary endpoints. (Full-analysis-set). **A** Proportion of participants achieving HbA1c target ≤ 6.5% in double-blind stage. and proportion of participants achieving HbA1c target < 7.0% in double-blind stage. **B** FBG change in double-blind stage. Data are mean and error bars are SEs. **C** FBG change in double-blind stage. Data are mean and error bars are SEs

Compared with subjects with placebo, significantly more patients with fotagliptin and alogliptin achieved the HbA1c targets (< 7.0% and ≤ 6.5%) after 24 weeks of treatment: 20.7%, 20.0% and 4.4% patients achieved HbA1c ≤ 6.5% in the fotagliptin group, alogliptin group and placebo group, respectively; 37.0%, 35.5% and 15.5% patients achieved HbA1c < 7.0% in the fotagliptin group, alogliptin group and placebo group, respectively (Fig. 3A). The proportion of patients achieved the HbA1c targets was similar between fotagliptin and alogliptin treatment groups at Week 24.

In contrast to placebo, fotagliptin and alogliptin resulted in significantly greater decrease in mean FBG after 24 weeks of treatment (Fig. 3B). Mean weight loss did not change substantially during the whole 52 weeks of treatment and no more than 1 kg of weight change was found in any group (Fig. 3C). During the whole 52 weeks of treatment, the overall incidence of hypoglycemia was relatively low (1.0% [2/211] for fotagliptin group, 1.0% [1/99] for alogliptin group and 3.8% [4/106] for placebo group).

Changes in additional efficacy measures from baseline to Week 24 and Week 52 are provided in Table 2. The assessment of β-cell function from baseline to Week 24 showed that both fotagliptin and alogliptin were associated with significant improvements in homoeostasis model assessment of β-cell (HOMA-β) compared with baseline, but no treatment related differences were recorded for HOMA index of insulin resistance (HOMA-IR), or fasting insulin concentration. Meanwhile, fotagliptin was associated with significant improvements in fasting C-peptide concentration compared with baseline. No significant changes in the aminotransferase and lipid profiles were observed in the three groups. DPP-4i may have a small effect on exocrine gland of pancreas as the serum amylase and lipase levels showed a minor increase across the fotagliptin, alogliptin and placebo groups from baseline to Week 52. Moreover, serum creatinine increased slightly in fotagliptin group.

**Table 2:** Change in additional secondary measures of efficacy from baseline to week 24 and week 52

	Mean change from baseline to week 24			
	Fotagliptin group	Alogliptin group	Placebo group	P
Fasting insulin (μIU/ml)	0.73(-0.13,1.59)	0.44(-0.51,1.38)	-0.11(-1.18,0.96)	0.671
Fasting C-peptide (pmol/L)	0.13(0.03,0.23)	0.08(-0.03,0.19)	-0.05(-0.16,0.06)	0.146
HOMA-β	11.00(7.46,14.55)	7.21(3.39,11.02)	4.61(0.66,8.56)	0.066
HOMA-IR	-0.12(-0.58,0.33)	-0.16(-0.63,0.30)	-0.46(-0.99,0.06)	0.980
ALT (U/L)	-2.26(-3.64,-0.89)	-2.31(-4.17,-0.44)	4.82(-5.99,15.63)	0.820
AST (U/L)	-1.01(-1.83,-0.19)	-0.81(-2.10,0.48)	1.94(-3.79,7.68)	0.836
Serum creatinine (umol/L)	2.28(1.19,3.37)	-0.50(-1.96,0.96)	0.76(-0.97,2.49)	0.018
eGFR (ml/min per 1.73 m2)	-1.83(-2.85,-0.80)	0.90(-0.39,2.19)	-0.96(-2.64,0.73)	0.018
LDL-C (mmol/L)	0.02(-0.07,0.11)	-0.04(-0.16,0.09)	0.06(-0.07,0.19)	0.225
TG (mmol/L)	0.15(-0.08,0.37)	0.23(0.01,0.44)	-0.12(-0.28,0.04)	0.261
Serum amylase (U/L)	4.10(1.89,6.30)	1.92(-1.36,5.20)	0.52(-2.27,3.32)	0.107
Serum lipase (U/L)	6.35(2.02,10.67)	6.14(3.03,9.25)	0.67(-1.29,2.63)	0.003
	Mean change from baseline to week 52			
	Fotagliptin group	Alogliptin group	Control group	P
Fasting insulin (μIU/ml)	-0.52(-1.29,0.26)	0.24(-0.95,1.43)	0.16(-1.12,1.44)	0.661
Fasting C-peptide (pmol/L)	0.07(-0.01,0.15)	0.14(-0.00,0.28)	0.14(-0.02,0.29)	0.640
HOMA-β	3.76(0.56,6.96)	4.42(-0.08,8.93)	6.94(1.96,11.92)	0.648
HOMA-IR	-0.44(-0.91,0.04)	-0.20(-0.79,0.40)	-0.36(-1.03,0.31)	0.630
ALT (U/L)	-0.37(-2.15,1.41)	-1.25(-3.35,0.86)	2.28(-1.59,6.14)	0.697
AST (U/L)	0.22(-0.75,1.19)	-0.53(-1.77,0.71)	1.77(-0.22,3.76)	0.443
Serum creatinine (umol/L)	3.15(1.60,4.71)	0.67(-1.17,2.51)	2.66(0.84,4.48)	0.144
eGFR (ml/min per 1.73 m^2^)	-2.34(-3.54,-1.14)	-0.62(-2.40,1.15)	-2.24(-3.88,-0.59)	0.184
LDL-C (mmol/L)	0.03(-0.07,0.13)	-0.04(-0.18,0.11)	0.04(-0.11,0.20)	0.676
TG (mmol/L)	0.27(-0.05,0.58)	0.22(0.02,0.42)	-0.06(-0.32,0.21)	0.111
Serum amylase (U/L)	4.59(3.07,6.10)	3.16(0.79,5.54)	5.62(2.51,8.72)	0.744
Serum lipase (U/L)	6.83(4.30,9.35)	6.37(3.56,9.18)	6.65(3.90,9.41)	0.113

Table 3 summarized the clinical adverse events, which showed that fotagliptin and alogliptin were well tolerated in all treatment groups. No deaths were reported during the study in all treatment groups. No serious adverse events occurred in more than 2% of the patients in either treatment group. All adverse events occurred in 4% of participants or fewer, and adverse events occurred infrequently in all groups in double-blind period (3.1% for fotagliptin group, 3.6% for alogliptin group and 6.2% for placebo group). Adverse events that were considered to be treatment-emergent (TEAEs) and occurred in more than 5% of any treatment group are shown in Table 3. The most commonly reported TEAE was lipase increased, reported by 7 (3.1%), 10 (9.1%), 2 (1.8%) of patients in the fotagliptin, alogliptin and placebo groups, respectively. Statistically significant difference in the occurrence of lipase increased was observed between the alogliptin and placebo groups. Minor hypoglycemia was reported in participants treated with fotagliptin (2 [0.9%]) and alogliptin (0 [0.0%]) in double-blind period. The occurrence of hypoglycemia was similar in all treatment groups in the extended period. Unexpectedly, seven patients in the placebo group experienced hypoglycemia in double-blind period. All hypoglycemia events were considered mild to moderate in intensity with no need for assistance from others.

**Table 3:** Summary of clinical adverse events in the treated set of safety population

		Fotagliptin group	Alogliptin group	Placebo group	P
Serious adverse events that were present in more than 2% of the patients in either trial group, n (%)					
Double-blind stage	-	-	-	-	-
Extended stage	-	-	-	-	-
Adverse events that were considered to be related to therapeutic drug or placebo in more than 5% of the patients in either trial group, n (%)					
Double-blind stage	Lipase increased	7 (3.1)	10 (9.1)^#^	2 (1.8)	0.0217
Extended stage	-	-	-	-	-
Any other safety events of special interest, n (%)					
Double-blind stage	Hypoglycemia	2 (0.9)^*^	0 (0.0)^#^	7 (6.2)	0.0038
	Myocardial ischaemia	2 (0.9)	2 (1.8)	2 (1.8)	0.6441
	Arteriosclerosis coronary artery	2 (0.9)	0 (0.0)	1 (0.9)	1.0000
	Acute coronary syndrome	1 (0.4)	0 (0.0)	0 (0.0)	1.0000
Extended stage	Hypoglycemia	2 (1.0)	1 (1.0)	4 (3.8)	0.1604
	Myocardial ischaemia	2 (1.0)	0 (0.0)	2 (1.9)	0.5587
	Angina pectoris	1 (0.5)	0 (0.0)	0 (0.0)	1.0000
	Arteriosclerosis coronary artery	1 (0.5)	0 (0.0)	1 (1.0)	1.000
Any adverse events more than 5% in either trial group					
Double-blind stage	Dyslipidemia	28 (12.3)	17 (15.5)	15 (13.3)	0.7600
	Hyperuricemia	18 (7.9)	7 (6.4)	3 (2.7)	0.1622
	Urinary tract infection	13 (5.7)	9 (8.2)	7 (6.2)	0.7051
	Upper respiratory tract infection	14 (6.1)	8 (7.3)	6 (5.3)	0.8416
	Lipase increased	8 (3.5)	11 (10.0)^##^	2 (1.8)	0.0081
	Hypoglycemia	2 (0.9)^*^	0 (0.0)^#^	7 (6.2)	0.0038
Extended stage	Dyslipidemia	17 (8.2)	10 (10.2)	5 (4.8)	0.3880
	Hyperuricemia	4 (1.9)	7 (7.1)	7 (6.7)	0.0347
	Abnormal hepatic function	6 (2.9)	4 (4.1)	6 (5.8)	0.4256

1 The safety population was defined as patients who took at least one dose of therapeutic drug or placebo

2 Data are number (% of total participants in treatment group)

3 *P < 0.05 compared with control group; ^#^*P* < 0.05; ^##^*P* < 0.01 compared with control group

4 This table lists serious adverse events that were present in more than 2% of the patients in either trial group, adverse events that were considered to be related to therapeutic drug or placebo in more than 5% of the patients in either trial group, and any other safety events of special interest

## Discussion

The multicenter clinical study showed that in T2DM patients inadequately controlled with diet and exercise intervention, fotagliptin 12 mg once daily for 24 weeks provided superior glycemic control compared with placebo, as assessed by reductions in HbA1c and FBG. No clinically significant difference in the improvements in clinical response of glycemic control was observed between the fotagliptin group and the alogliptin group.

HbA1c represents the most powerful predictor of diabetes related outcomes and mortality [15, 16]. The phase 3 randomized, double-blind, placebo-controlled clinical study of fotagliptin achieved its primary efficacy endpoint. In treatment-naive T2DM patients uncontrolled with diet and exercise intervention, fotagliptin monotherapy achieved clinically and significant meaningful amelioration in the percentage of patients reached HbA1c targets (< 7.0% and ≤ 6.5%). Current guidelines suggest that should be set for most adults with diabetes, as concerns remain that aggressive hypoglycemic treatment may increase the risk of diabetic complications [17, 18]. Fotagliptin demonstrated a favorable safety profile during the 52 weeks treatment, and there were no serious hypoglycemia or severe adverse events that required medical assistance. Clinically significant hypoglycemia occurred only in two patients (0.9%) treated with fotagliptin over 24 weeks, and the events were mild in nature. The incidence of hypoglycemia reported in this study is consistent with other studies to investigate the efficacy and safety of DPP-4 inhibitors therapy [19, 20]. Mild but not clinically significant weight change was observed in all treatment groups, including placebo. These may be attributed to the pharmacokinetic and pharmacodynamic results of both fotagliptin and alogliptin. One recent study in Chinese patients with T2DM indicated that fotagliptin could increase plasma GLP-1 concentration while DPP-4 inhibition was continuously maintained at a steady state [12]. Still, relevant results were based on the design of the study and patients were closely monitored to ensure that they followed the diet and exercise guidelines of the trail. Given an increasing use of DPP-4 inhibitors to treat patients with T2DM in the real-world setting, potential pancreatitis risk has been of concern because of its consequent pharmacological mechanism of the pancreas [7, 21]. Overall, no pancreatitis occurred during the whole treatment period. As expected, slight increases of amylase and lipase levels were observed with fotagliptin and alogliptin from Week 4 to Week 24, and this trend gradually decreased in the later period of the trail. No significant change from baseline in pancreatic enzymes was observed in placebo group.

Fotagliptin is a novel highly selective DPP-4 inhibitor under clinical development for the increased levels of intact bioactive GIP and GLP-1. Based on previous studies, fotagliptin could increase active GLP-1 concentrations and have no obvious influence on DPP-8 and DPP-9, thus making it safer to treat T2DM [13]. To our knowledge, this was the first adequately powered trial to investigate the efficacy and safety profiles of fotagliptin in patients with uncontrolled T2DM. The reductions in HbA1c with fotagliptin were much the same as those with alogliptin in the double-blind period. In addition, the study results did not demonstrate any benefit of fotagliptin for most additional secondary efficacy measures except for fasting C-peptide, which we suspected that may be related to the relieved glycotoxicity in some patients. Serum creatinine increased slightly with fotagliptin and differences were small but significant for three groups in the double-blind period. Nevertheless, such difference no longer existed in the extended treatment period. In the double-blind period, the proportion of subjects receiving rescue therapy in fotagliptin group was significantly lower than that in the placebo group, but the proportion increased in the extended period for both the fotagliptin group and alogliptin group. Interestingly, after the conversion to fotagliptin in the extended period, the proportion of subjects receiving rescue therapy in the original placebo group increased, although only 24 weeks of delayed hypoglycemic treatment. Therefore, in order to obtain long-term benefits, it is reasonable for patients with T2DM to start hypoglycemic therapy as soon as possible after the diagnosis of diabetes.

However, limitations existed in the present trial. First of all, there is a potential limitation in interpreting effect of fotagliptin compared with placebo on risk of major cardiovascular (CV) events, as the study duration may be too short to modulate CV related clinical outcomes. Four previous CV outcome trials of DPP-4 inhibitors have demonstrated a non-inferior risk of a composite CV outcome when compared with placebo [22–25]. Secondly, the follow-up duration was relatively short at approximately 52 weeks. No other benefits and risks of long-term treatment with fotagliptin were evaluated. Third, although the comparison between fotagliptin and placebo in previously untreated diabetes patients who are not controlled with diet and exercise provided the exact glucose-lowering of the efficacy and safety of fotagliptin, we should notice that treatment with metformin is still the first-line therapy for patients with T2DM [26].

## Conclusion

Overall, the trial demonstrated improvement in glycemic control for fotagliptin monotherapy with 12 mg once daily in previously untreated T2DM patients uncontrolled with lifestyle intervention. Furthermore, fotagliptin treatment was not associated with greater risk of hypoglycemia episodes and weight gain, as compared with placebo and alogliptin. Thus, fotagliptin is a potential new approach for the treatment of T2DM patients who are inadequately controlled with diet and exercise intervention.

### Supplementary Information

**Additional file 1:** **Supplementary Table 1.** Full inclusion and exclusion criteria. **Supplementary Figure 1.** Clinical study design. **Supplementary Figure 2.** Differences in primary clinical end points (Full-analysis-set; Center effect) HbA1c values change in double-blind stage. Data are mean and error bars are SEs. The ETD and corresponding 95% CI were estimated using an ANCOVA with center effect in the FAS. Last observation carried forward imputation was used for missing values. **Supplementary Figure 3.** Differences in primary clinical end points (Full-analysis-set; Observed data) HbA1c values change in double-blind stage. Data are mean and error bars are SEs. The ETD and corresponding 95% CI were estimated using an ANCOVA without missing-value imputation in the FAS, including observation data after rescue. **Supplementary Figure 4.** Differences in primary clinical end points (Full-analysis-set; MMRM) HbA1c values change in double-blind stage. Data are mean and error bars are SEs. The ETD and corresponding 95% CI were estimated using a mixed model of repeated measure (MMRM) without missing-value imputation in the FAS. **Supplementary Figure 5.** The multiplicity strategy for primary end point was the fixed-sequence test.

## Data Availability

The data that support the findings of this study are available on request from the corresponding authors after the completion of the study. The data are not publicly available due to privacy or ethical restrictions.

## Abbreviations

ANCOVA: Analysis of covariance model
CV: Cardiovascular
DPP-4i: Dipeptidyl peptidase-4 inhibitors
ETD: Estimated treatment difference
FBG: Fasting blood glucose
HbA1c: Hemoglobin A1C
HOMA-β: Homoeostasis model assessment of β-cell
HOMA-IR: Homoeostasis index of insulin resistance
IC50: Half of maximal inhibitory concentration
IWRS: Interactive web response system
IMP: Investigational medicinal products
GLP-1: Glucagon-like peptide-1
LOCF: The last observation carried forward
T2DM: Type 2 diabetes mellitus
t_max_: Maximum serum concentration
TEAE: Treatment-emergent adverse event
SD: Standard deviation
SE: Standard error
95% CI: 95% Confidence interval
