# Supplementary material for: Fotagliptin monotherapy with alogliptin as an active comparator in patients with uncontrolled type 2 diabetes mellitus: a randomized, multicenter, double-blind, placebo-controlled, phase 3 trial
Source: BMC Med. 2023 Oct 9;21:388. doi: 10.1186/s12916-023-03089-x (PMC10563289; doi:10.1186/s12916-023-03089-x)
Supplement: Supplementary file 1 — Additional file 1: Supplementary Table 1. Full inclusion and exclusion criteria. Supplementary Figure 1. Clinical study design. Supplementary Figure 2. Differences in primary clinical end points (Full-analysis-set; Center effect) HbA1c values change in double-blind stage. Data are mean and error bars are SEs. The ETD and corresponding 95% CI were estimated using an ANCOVA with center effect in the FAS. Last observation carried forward imputation was used for missing values. Supplementary Figure 3. Differences in primary clinical end points (Full-analysis-set; Observed data) HbA1c values change in double-blind stage. Data are mean and error bars are SEs. The ETD and corresponding 95% CI were estimated using an ANCOVA without missing-value imputation in the FAS, including observation data after rescue. Supplementary Figure 4. Differences in primary clinical end points (Full-analysis-set; MMRM) HbA1c values change in double-blind stage. Data are mean and error bars are SEs. The ETD and corresponding 95% CI were estimated using a mixed model of repeated measure (MMRM) without missing-value imputation in the FAS. Supplementary Figure 5. The multiplicity strategy for primary end point was the fixed-sequence test. [file 12916_2023_3089_MOESM1_ESM.pdf]

Supplementary information

Supplementary Table 1 Full inclusion and exclusion criteria

1. Inclusion criteria

Patients should meet all the following criteria.

(1) Patients with type 2 diabetes WHO meet the diagnostic criteria for diabetes issued by the World Health Organization (WHO) in 1999;

(2) Male or female ≥18 years old and ≤75 years old at the time of signing the informed consent;

(3) Body mass index: 19 kg/m^2 ≤ BMI ≤ 35 kg/m^2, [BMI= weight (kg)/height ^{2} (m^2)];

(4) Poor blood glucosez control after diet and exercise, and meet the following requirements:

i. Diet and exercise therapy for at least 4 weeks before screening;

ii. The total number of days receiving any hypoglycemic drug therapy within 8 weeks before screening was no more than 7 days;

(5) Glycosylated hemoglobin (HbA1c) at screening visit: 7.5%≤HbA1c≤10.5% (measured by the research center); at random visit: 7.0%≤HbA1c≤10.0% (measured by the central laboratory);

(6) Fasting blood glucose ≤13.9mmol/L during screening and randomization;

(7) Able to understand the procedures and methods of the study, willing to strictly follow the clinical trial protocol to complete the study, and voluntarily sign the informed consent.

2. Exclusion criteria

Patients should not meet any of the following criteria.

(1) Patients with medication compliance < 80% or > 120% during the run-in period;

(2) Use other hypoglycemic drugs except experimental drugs during the run-in period;

(3) Patients may have any contraindications, allergies, or hypersensitivity to fotagliptin (including the study drug and placebo) or its excipients, DPP4 drugs, metformin;

(4) Prior to screening, any of the following endocrine-related history or evidence:

i. Diabetes other than type 2 diabetes, such as type 1 diabetes, monogene mutation diabetes, diabetes due to pancreatic damage or secondary diabetes, such as Cushing's

syndrome or acromegaly;

ii. Patients with a history of diabetic ketoacidosis, hyperglycemia hyperosmolar state, lactic acidosis and other acute complications of diabetes within 6 months before screening;

iii. A history of severe hypoglycemia (such as lethargy, disturbance of consciousness, gibberish, or even coma caused by hypoglycemia), or a history of severe unconscious hypoglycemia;

iv. Severe chronic complications of diabetes;

v. Other serious endocrine diseases, such as hyperthyroidism, hypothyroidism, hypercortisolism, multiple endocrine tumors, etc.;

（5）History or evidence of any of the following diseases prior to screening:

i. Unstable angina, stroke or transient ischemic attack, myocardial infarction, coronary artery bypass grafting or percutaneous coronary intervention in the 6 months prior to screening (diagnostic angiography was allowed);

ii. Prescreening decompensated heart failure (NYHA cardiac function grades III and IV, persistent and clinically significant arrhythmias;

iii. A history of hypertension with a stable dose (at least 4 weeks) of antihypertensive drugs followed by sitting systolic blood pressure (SBP) ≥160mmHg and/or sitting diastolic blood pressure (DBP) ≥100mmHg;

iv. History of acute and chronic pancreatitis, history of symptomatic gallbladder or pancreatic injury, and other high-risk factors that may lead to pancreatitis;

v. Severe trauma or acute infection that may affect blood glucose control within 4 weeks prior to screening;

vi. Obvious hematological diseases or any diseases that cause hemolysis or erythrocyte instability, including but not limited to: hemolytic anemia, iron deficiency anemia, aplastic anemia, massive blood loss or blood transfusion, uremia, chronic malaria, splenectomy, reticulocytosis, etc.;

vii. A history of malignant tumor within 5 years prior to screening;

viii. A history of severe chronic gastrointestinal disease or treatment that may interfere with

drug absorption, such as gastrointestinal surgery; Immunocompromised subjects who have received organ transplants (keratoplasty is permitted) or have been diagnosed with human immunodeficiency virus infection;

ix. Mental or neurological disease, unwillingness to communicate or a language barrier, inability to fully understand and collaborate；

（6） Any of the following drugs or treatments were used before screening:

i. Use of weight-loss drugs or surgery that can lead to weight instability within 3 months prior to screening;

ii. Received corticosteroid therapy (excluding topical or inhaled preparations) within 3 months prior to screening;

iii. Treated with growth hormone within 6 months prior to screening;

iv. Use of any drug that may interfere with the interpretation of efficacy and safety data, or use of any drug or Chinese herb known to have common toxic effects on major organs, as determined by the investigator, during the 8 weeks prior to screening；

（7） Any of the laboratory test indicators during screening meet the following criteria:

i. ALT > 2 times the upper limit of normal value;

ii. AST > 2 times the upper limit of normal value;

iii. Total bilirubin > 1.5 times the upper limit of normal value;

iv. Serum creatinine ≥1.5mg/dL for males and 1.4mg/dL for females;

v. eGFR < 60mL/min/1.73m2;

vi. Creatine kinase > 3 times the upper limit of normal;

vii. Triglyceride > 5mmol/L;

viii. TSH < 0.1mU/L;

ix. Any clinically significant abnormal laboratory values that the investigator believes may interfere with the evaluation of efficacy and/or safety data for this study;

（8） During screening, degree II or III atrioventricular block, long QT syndrome, or QTc > 500ms occurred in 12-lead ECG without pacemaker installation;

（9） A history of substance abuse or alcohol abuse in the six months preceding screening (14 units of alcohol per week: 1 unit = 285mL beer, 25mL spirits, or 100mL wine);

(10) Participated in any other clinical trials for drugs or medical devices within 3 months prior to screening;

(11) Received blood transfusion within 8 weeks prior to screening;

(12) Female subjects who are pregnant, lactating or have positive pregnancy test;

(13) The subjects or their partners do not want to take effective contraceptive measures during the trial or plan to have a family within 1 month after the end of the trial;

(14) Previously participated in other clinical studies of fotagliptin benzoate;

(15) Researchers directly associated with the study and/or their immediate family members. Immediate family members are spouses, parents, children or siblings (biological or adoptive);

(16) The investigator believes that there are any other factors that may affect the efficacy or safety evaluation of the study that are not suitable to participate in the clinical trial (including but not limited to the investigator's judgment that the subjects' compliance is poor, or their residence is too far away, and they cannot follow up on schedule, etc.).

Supplementary Figure Legends

Supplementary Figure 1. Clinical study design.

Supplementary Figure 2. Differences in primary clinical end points (Full-analysis-set; Center effect)

HbA1c values change in double-blind stage. Data are mean and error bars are SEs. The ETD and corresponding 95% CI were estimated using an ANCOVA with center effect in the FAS. Last observation carried forward imputation was used for missing values.

Supplementary Figure 3. Differences in primary clinical end points (Full-analysis-set; Observed data)

HbA1c values change in double-blind stage. Data are mean and error bars are SEs. The ETD and corresponding 95% CI were estimated using an ANCOVA without missing-value imputation in the FAS, including observation data after rescue.

Supplementary Figure 4. Differences in primary clinical end points (Full-analysis-set; MMRM)

HbA1c values change in double-blind stage. Data are mean and error bars are SEs. The ETD and corresponding 95% CI were estimated using a mixed model of repeated measure (MMRM) without missing-value imputation in the FAS.

Supplementary Figure 5. The multiplicity strategy for primary end point was the fixed-sequence test.

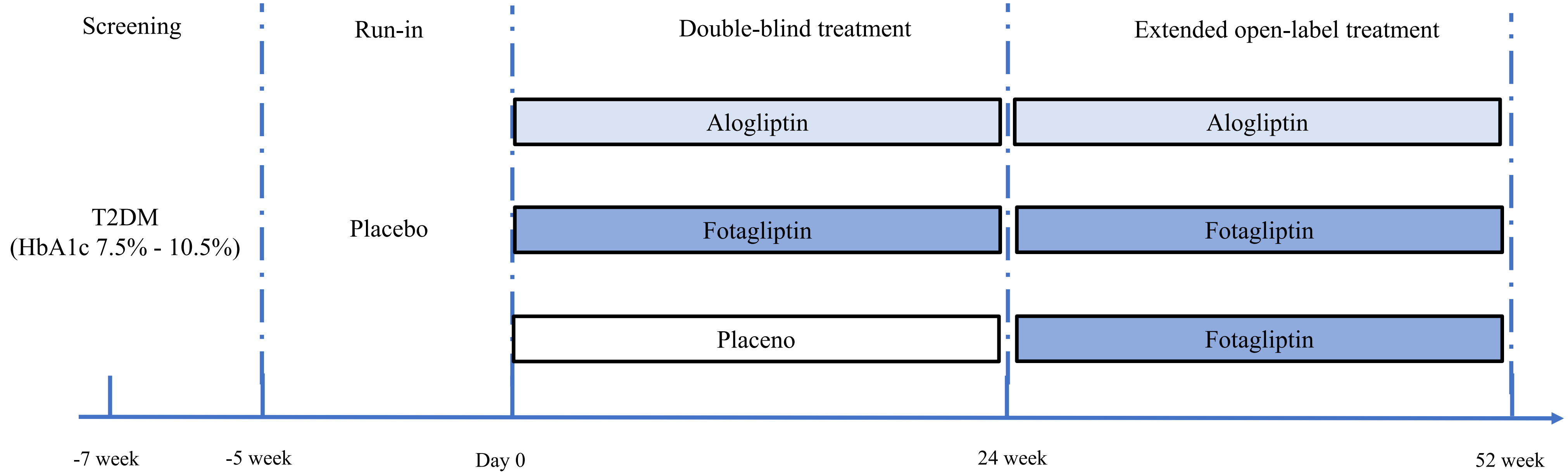

Supplementary Figure 1

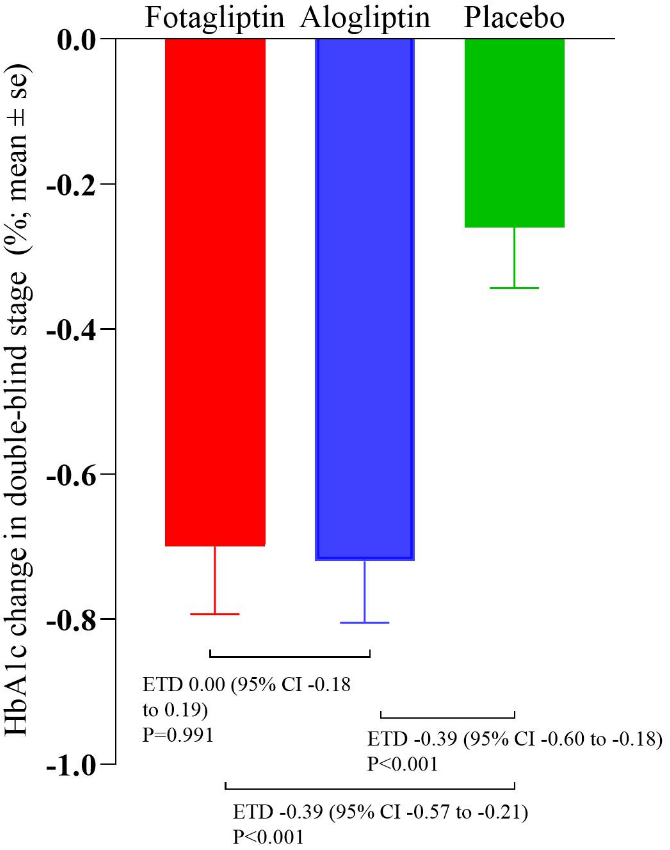

Supplementary Figure 2

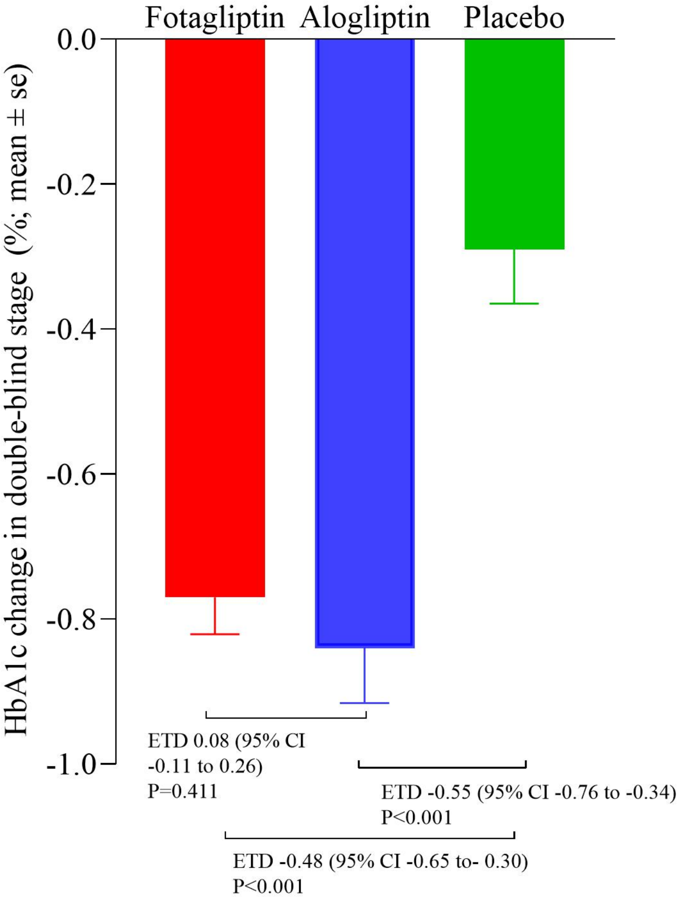

Supplementary Figure 3

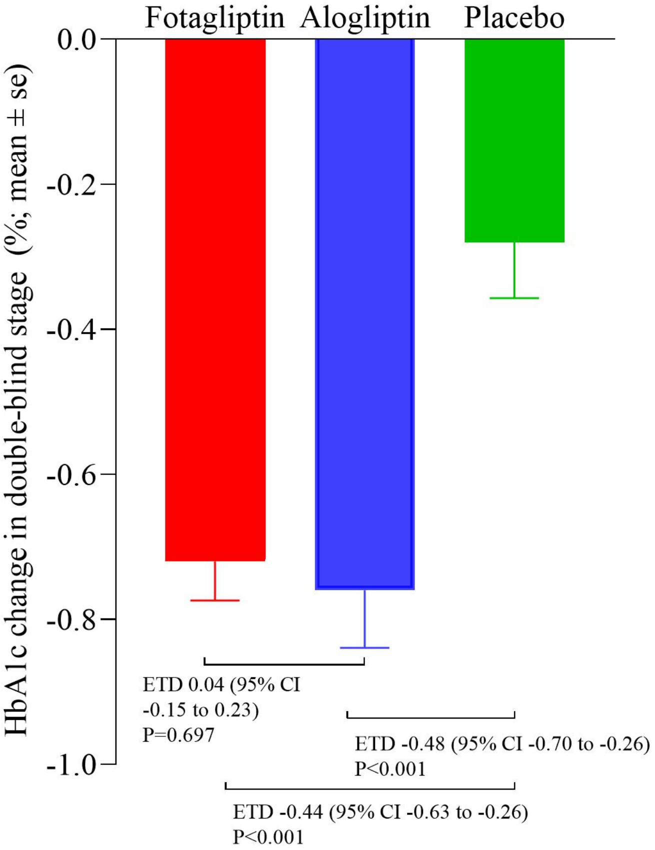

Supplementary Figure 4

Step 1 is the superiority comparison between fotagliptin vs placebo, if significant （P<0.05） then do step 2, otherwise stop. Step 2 is the superiority comparison between alogliptin vs placebo, if significant （P<0.05） then do step 3, otherwise stop. Step 3 is the non-inferiority comparison between fotagliptin and alogliptin.

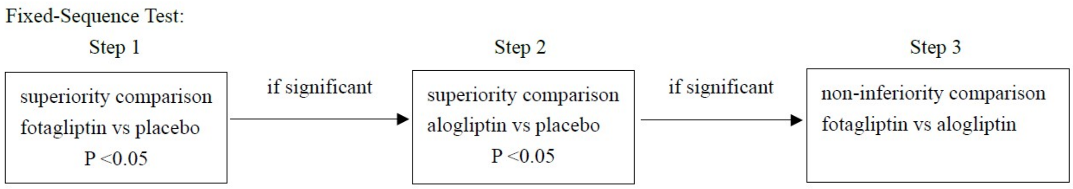

Supplementary Figure 5
